# Exploring the impact of telehealth on the work of frontline health professionals within the Whole System Demonstrator study

**Published:** 2012-06-15

**Authors:** Virginia MacNeill, Ray Fitzpatrick

**Affiliations:** Barts and the London School of Medicine and Dentistry, Queen Mary University of London, UK; Department of Public Health, University of Oxford, UK

**Keywords:** Whole System Demonstrator, telehealth, health professionals, qualitative

## Abstract

**Introduction:**

A central aim of the Whole System Demonstrator (WSD) programme is to find out how telehealth can help people manage their own long-term health conditions and to evaluate the potential benefits of this technological intervention within a randomised control trial. However, the adoption of this intervention at local level will entail significant changes in practice of care, in the division of health care work and in traditional professional-patient relationships and so will be contingent on the support of frontline health professionals directly responsible for its delivery. This was the rationale behind this theme 4 sub study within the Whole System Demonstrator evaluation study.

**Aims and objectives:**

To examine health professionals’ views and experiences of implementing telehealth and their retrospective judgements of barriers and facilitators to its success.

**Methods:**

We undertook a 2-year qualitative longitudinal study. The method of data collection was semi-structured in-depth interviews with a purposive sample of frontline health professionals. The first year comprised baseline qualitative interviews with key health professionals directly involved in the delivery of telehealth in three Whole System Demonstrator sites: the London Borough of Newham, Cornwall and Kent. Interview participants included community matrons, teleheath monitoring nurses and general practitioners who deliver care to patients with long-term conditions. Follow-up interviews were conducted around one year later, prior to the end of the study and all interview data were thematically analysed and managed by NVivo.

**Results:**

Currently under embargo by DH.

**Conclusions:**

Currently under embargo by DH.

